# Combination Therapy with Pyridoxine and Arginine Supplementations along with a Lysine-Restricted Diet in Individuals with Pyridoxine-Dependent Epilepsy: A Comprehensive Systematic Review

**DOI:** 10.1016/j.cdnut.2025.107504

**Published:** 2025-07-08

**Authors:** Ali Jafari, Mohammad Mehdi Abbasi, Hamid Abbasi, Sama Rahnemayan, Farnush Bakhshimoghaddam, Saeid Doaei

**Affiliations:** 1 Student Research Committee, Department of Community Nutrition, Faculty of Nutrition Sciences and Food Technology, National Nutrition and Food Technology Research Institute, Shahid Beheshti University of Medical Sciences, Tehran, Iran; 2 Systematic Review and Meta-analysis Expert Group, Universal Scientific Education and Research Network, Tehran, Iran; 3 Student Research Committee, Faculty of Nutrition and Food Technology, Shahid Beheshti University of Medical Sciences, Tehran, Iran; 4 Student Research Committee, Tabriz University of Medical Sciences, Tabriz, Iran; 5 Neurosciences Research Center (NSRC), Tabriz University of Medical Sciences, Tabriz, Iran; 6 Department of Nutrition, School of Allied Medical Sciences, Ahvaz Jundishapur University of Medical Sciences, Ahvaz, Iran; 7 Department of Community Nutrition, Faculty of Nutrition and Food Technology, National Nutrition and Food Technology Research Institute, Shahid Beheshti University of Medical Sciences, Tehran, Iran; 8 Unit of Nutrition and Cancer, Cancer Epidemiology Research Program, Catalan Institute of Oncology, Bellvitge Biomedical Research Institute (IDIBELL), L’Hospitalet de Llobregat, Barcelona, Spain

**Keywords:** epilepsy, pyridoxine, arginine, lysine, diet

## Abstract

**Background:**

Pyridoxine-dependent epilepsy (PDE) is identified as a rare neurometabolic disease marked by biallelic pathogenic mutations of the ALDH7A1 gene. A combination therapy involving pyridoxine, arginine supplementation (AS), and a lysine-restricted diet (LRD) was frequently reported to effectively improve PDE through reducing neurotoxic lysine metabolites, improving seizure management, and enhancing neurodevelopmental outcomes.

**Objectives:**

The study sought to investigate the effects of mono-(pyridoxine), dual- (pyridoxine combined with AS or LRD), and triple-therapy approaches in individuals diagnosed with PDE.

**Methods:**

An extensive search was carried out across international databases, comprising Scopus, Embase, Web of Science, PubMed, and Google Scholar, to find relevant publications published before 12 November, 2024. The methodological quality assessment of chosen articles was evaluated utilizing the Newcastle-Ottawa Scale and the Joanna Briggs Institute tool.

**Results:**

Among 2097 studies reviewed, 38 met inclusion criteria, covering treatment methods for individuals with PDE including monotherapy (22 articles), dual therapy (9 articles), and triple therapy (7 articles). The results indicated that pyridoxine monotherapy is a highly effective first-line treatment in PDE that improves seizure control with minimal cognitive decline. Combining pyridoxine with an LRD or AS targets metabolic issues, reducing neurotoxic metabolites and enhancing cognitive and motor functions. Early triple therapy, within the first 6 months of life, exhibited significant benefits for seizure management and cognitive performance in patients with PDE.

**Conclusions:**

In summary, administration of pyridoxine is highly effective, particularly when combined with AS and an LRD. Triple therapy illustrates promise for improved seizure control and cognitive function, especially when initiated early. Further research is warranted.

## Introduction

**FIGURE 1:**
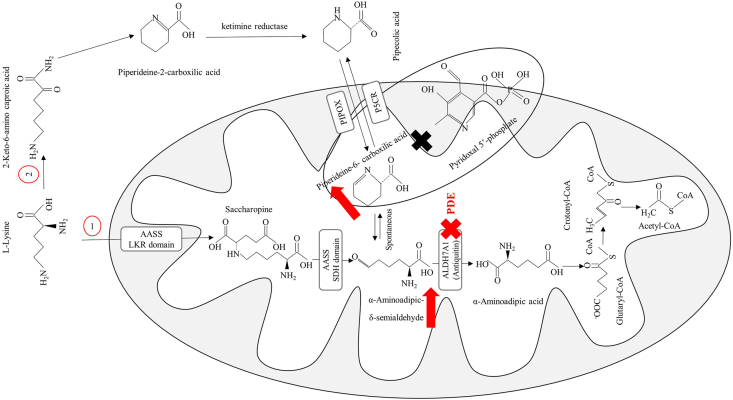
Saccharopine (1) and pipecolic acid (2) pathways are crucial for L-lysine catabolism. The L-pipecolic acid pathway involves the lysine-ketoglutarate reductase (LKR) and saccharopine dehydrogenase (SDH) domains of the bifunctional aminoadipic semialdehyde synthase (AASS) enzyme. Deficiency of antiquitin, encoded by the ALDH7A1 gene, results in pyridoxine-dependent epilepsy (PDE), causing an accumulation of α-aminoadipic-δ-semialdehyde (AASS) and piperideine-6-carboxilic acid (P6C).

Pyridoxine-dependent epilepsy (PDE, OMIM code: #266100), also identified as pyridoxine-dependent developmental and epileptic encephalopathy, is a rare neurometabolic disease inherited from biallelic deleterious mutations in ALDH7A1 gene, situated at the 5q32.3 locus [1]. The dysfunction of α**-**aminoadipic semialdehyde dehydrogenase (AASAHD), an enzyme responsible to the conversion of α**-**aminoadipic semialdehyde to α**-**aminoadipic acid, is the primary factor contributing to this condition [2]. The global prevalence of PDE varies significantly, with estimates indicating a frequency between ∼1 in 65,000 to 1 in 250,000 live births [3]. A deficiency in AASAHD results in the pathological buildup of α**-**aminoadipic semialdehyde (AASA), its cyclic derivative Δ1-piperideine-6-carboxylate (P6C), and pipecolic acid (PA) within urine, cerebrospinal fluid (CSF), and plasma (Figure 1). The overabundance of P6C is thought to sequester the bioactive variant form of vitamin B6 (VB6), pyridoxal 5’-phosphate, which is hypothesized to precipitate intractable seizures observed in individuals with PDE [4]. Furthermore, anomalies in neuronal migration and various cerebral malformations including hydrocephalus and irregularities in the white matter, corpus callosum, and posterior fossa have been documented in conjunction with PDE, implicating the ALDH7A1 gene, which encodes AASAHD, in the processes of neuronal development [5].

The prototypical manifestation of PDE is marked by the onset of seizures during the neonatal period, which exhibits resistance to standard antiepileptic pharmacotherapy. However, these seizures often demonstrate a remarkable responsiveness to a rapid intervention involving a single intravenous administration of pyridoxine (with pharmacologic doses ranging from 50 to 100 mg) as a first-line treatment, leading to a cessation of seizure activity and restoration of electroencephalographic (EEG) normalcy in roughly 85% of affected individuals [6]. Nevertheless, despite achieving satisfactory seizure control, ∼75% of individuals diagnosed with PDE experience considerable developmental delays and intellectual disabilities when treated exclusively with pyridoxine monotherapy [7,8]. Notably, despite prompt diagnosis and ideal management of seizures, considerable cognitive impairments have been detected in both pediatric and adult populations. This indicates that pyridoxine supplementation alone may be inadequate for addressing the comprehensive neurological manifestations of the disorder [8,9]. Ample evidence obtained from case series and case reports illustrates that triple therapy utilizing pyridoxine, arginine supplementation (AS) along with a lysine-restricted diet (LRD) has a beneficial impact on neurocognitive improvements [[10], [11], [12], [13], [14]]. Triple therapy was reported to have robust tolerability and adherence, with no adverse events documented [11]. Furthermore, significant improvement in cognitive status was observed alongside a marked decrease in seizure frequency and a reduction in PA levels, serving as a critical biomarker for the condition [11]. Adherence to the triple therapy was found to diminish AASA concentrations in both plasma and urine, as well as the concentrations of plasma P6C PA, and detectable neurocognitive improvement [10]. A notable clinical response was observed in a patient with seizure activity after the administration of triple therapy, which resulted in a marked reduction in seizure frequency. Concurrently, there was a meaningful decrease in urine PA concentrations post-therapy [12].

To provide a comprehensive overview of monotherapy, dual therapy, and triple therapy—including pyridoxine, AS, and LRD—in individuals diagnosed with PDE, we conducted an exhaustive systematic review of case reports, cohort studies, case series, and case-control studies. This review aimed to present overarching evidence regarding the efficacy and impact of these treatments in managing PDE.

## Methods

### Protocol and registration

This study was carried out in alignment with the PRISMA guidelines [15]. The study’s protocol was registered in the PROSPERO under ID: CRD42024590620.

### Literature search

A meticulous exploration of international electronic databases, encompassing Scopus, Embase, Web of Science, PubMed, and Google Scholar, was executed, including all available records up to 12 November, 2024. The systematic review’s search strategy was precisely formulated utilizing the following targeted keywords alongside Medical Subject Headings (MeSH) to enhance retrieval efficacy: (("Lysine"[MeSH Terms] AND "Diet"[MeSH Terms]) OR "Pyridoxine"[MeSH Terms] OR "Arginine"[MeSH Terms] OR ("Arginine"[Title/Abstract] OR "Pyridoxine"[Title/Abstract] OR "Vitamin B6"[Title/Abstract] OR ("Lysine"[Title/Abstract] AND "Diet"[Title/Abstract]))) AND ("Epilepsy"[MeSH Terms] AND ("Pyridoxine"[Title/Abstract] AND "Epilepsy"[Title/Abstract])). Additionally, a comprehensive review of the bibliographies of selected articles was undertaken to mitigate the possibility of neglecting critical literature. The sensitivity of the search protocol was increased through the incorporation of a wildcard operator “∗”. Detailed search strategies employed across the databases are accessible in Supplemental Appendix 1.

### Eligibility criteria

In this systematic review, articles were meticulously chosen according to the established inclusion criteria: *1*) full-text research publications using case control, cohort, case series, and case report, *2*) articles assessing the efficacy of monotherapy, dual therapy, and triple therapy comprising pyridoxine, AS in conjunction with an LRD in patients diagnosed with PDE. Exclusion criteria encompassed letters, literature reviews, commentaries, quasi-experimental research, and animal articles. This systematic review was structured according to the PICO framework: Population (P: individuals with PDE); Intervention (I: administration of pyridoxine, AS, and adherence to a diet restricted in lysine); Comparison (C: with or without control); Outcome (O: seizure frequency, cognitive function, seizure freedom, and the tolerability of treatment).

### Study selection

Duplicate entries were systematically eradicated utilizing EndNote software by a single investigator (AJ). Subsequently, the screening and selection evaluation phases, grounded in established eligibility criteria and the PICO framework, were carried out by 2 independent investigators (AJ and MMA). Any discrepancies or conflicts encountered during this process were resolved by the corresponding authors (HA and SD). Moreover, the references of all chosen publications were meticulously scrutinized to ensure that no pertinent articles were overlooked.

### Data extraction

An investigator (FB) meticulously gathered relevant data from the curated articles and organized it into a preformatted table created by one of the corresponding authors (HA). This table was then reviewed by the corresponding authors (HA and SD) for accuracy and completeness. The collected information included various elements such as the primary author’s name, the year of publication, sample size, study design, age demographics, number of males to females, symptoms and signs observed, clinical laboratory tests conducted, and their corresponding findings. The compiled data are presented in Table 1 [[10], [11], [12], [13], [14],[16], [17], [18], [19], [20], [21], [22], [23], [24], [25], [26], [27], [28], [29], [30], [31], [32], [33], [34], [35], [36], [37], [38], [39], [40], [41], [42], [43], [44], [45], [46], [47], [48]].

**TABLE 1:** Study characteristics of the selected articles.

Study	Publication year	Study design	Country	Sample size	Age	Male/female (n)	Symptoms and signs	Clinical laboratory test	Findings	Risk of bias
Falsaperla et al. [16]	2024	Retrospective cohort	Italy	3	4.3 y	NR	NR	Neurological test	The results indicate a link between diagnostic delay and negative neuromotor outcomes. This underscores the importance of early recognition and intervention in diagnosing and treating PDE.	Medium
Fortin et al. [17]	2023	Case report	United States	1	1 d	NR	Respiratory distress and episodes of rhythmic multifocal limb movements	Biochemical test, EEG, MRI	This study demonstrated that patients with PDE experienced gradual clinical improvement, which required ongoing treatment for several days. This highlights the importance of continuing vitamin B6 supplementation in suspected cases until confirmatory genetic testing is obtained or an alternate cause is identified.	Low
Chen et al. [18]	2022	Case series	China	4	5.5 mo	3 female, 1 male	Generalized tonic–clonic seizures,	MRI, EEG, clinical, and genetic tests	This study suggests that pyridoxine may be a promising adjunctive treatment option for patients with KCNQ2 epileptic encephalopathy.	Low
Amore et al. [19]	2022	Case series	Italy	1	7.5 y	1 female, 1 male	Clonic seizures, myoclonic seizures, perioral cyanosis	EEG, MRI	This study presented 2 cases of KCNQ2-related neonatal epilepsies, involving a 5-y-old male with a paternally inherited heterozygous mutation (c.1639C>T; p.Arg547Trp) and a 10-y-old female with a de novo heterozygous mutation (c.740C>T; p.Ser247Leu). Both children experienced improvements with VitB6 treatment.	Low
Alsubhi et al. [20]	2022	Case series	Canada	3	24 y	3 males	Nystagmus, dysarthria, truncal titubation, dysmetria, tremors, difficulty performing tandem gait, irritability, desaturations, metabolic lactic acidosis, tonic rigidity with gaze fixation, clonic jerking of the limbs, apnea	EEG, MRI, clinical and genetic test	This study underlines the importance of considering PLPBP mutations in individuals with partially B6-responsive seizures and highlights the presence of a founder effect in the French-Canadian population.	Low
Coughlin II et al. [10]	2022	Cohort	United States	60	6 mo	NR	NR	NR	The use of pyridoxine and LRTs showed a non-significant increase of 6.9 points on developmental testing compared with using pyridoxine alone. A subanalysis of 14 developmental testing scores from 8 participants revealed that using pyridoxine and LRTs in the first 6 mo of life was significantly associated with a 21.9-point increase in developmental testing.	Low
Bayat et al. [21]	2022	Cohort	Germany	7	12 y	1 female, 6 males	Bilateral tonic-colonic seizures, focal seizures, focal to bilateral tonic-clonic seizures, myoclonic seizures, tonic seizures	EEG, blood tests	This study found that >50% reduction in seizure frequency was observed in 2 out of 7 participants, and <50% reduction was seen in 3 out of 7. None of the participants achieved seizure freedom. Additionally, no significant changes in electrophysiological findings were noted in 6 of 7 participants who received pyridoxine or P5P when comparing baseline and follow-up EEGs.	Medium
Tseng et al. [22]	2022	Cohort	Netherlands	11	25.2 y	6 females, 5 male	NR	MRI, CTS, clinical, and genetic tests	Seizure control was achieved with pyridoxine monotherapy in 70% of cases, whereas 20% required additional common antiepileptic drugs. 10% did not achieve complete seizure control. Neurological symptoms were present in 90% of patients, including tremors in 40% of cases. 80% showed neuroimaging abnormalities, and 70% had intellectual disability.	High
Tseng et al. [23]	2022	Retrospective cohort	Netherlands	37	17.2 y	14 male, 23 female	NR	Neuroimaging and outcomes of PDE-ALDH7A1	Most siblings who received early treatment with pyridoxine alone showed better performance in fine motor skills than those who were treated later. Among the siblings who received pyridoxine along with adjunct LRT, most of those treated early showed better performance in overall neurodevelopment, cognition, and behavior/psychiatry as assessed clinically. Fourteen percent of the total group was assessed as normal in all domains.	Medium
Kim et al. [12]	2022	Case report	United States	1	3 d	1 male	Seizures, fever, respiratory distress, decreased cardiac function, and lactic acidosis	EEG, MRI, urine, blood, CSF cultures, genetic test	This case illustrates the diagnostic challenges in PDE, the utility of rapid whole-exome sequencing in such cases, and the response of urine pipecolic acid to therapy.	Low
Ryu et al. [24]	2022	Case report	Korea	1	9 y	1 male	Seizures, vomiting, poor general condition	EEG, clinical, and genetic tests	This study reports the case of a boy with intractable seizures related to ALDH7A1 mutations. Triple therapy successfully controlled his seizures and improved his behavior.	Low
Schmidt et al. [25]	2020	Case series	Canada	5	23.6 y	5 males	NR	NR	Results indicate that a daily intake of 300–600 mg/kg of L-arginine HCl and lysine, within the DRI limits, is necessary to decrease the absorption of lysine in the digestive system and its overall oxidation.	Low
Kava et al. [26]	2020	Case report	Australia	1	4 y	1 female	Lethargy, irritability, vomiting, abdominal distension, seizures and poor feeding	EEG, MRI, clinical and genetic test	This study demonstrates clinical and biochemical data obtained from a patient with antiquitin deficiency. In addition to standard treatment with pyridoxine, the patient has been managed with a lysine-restricted diet since the neonatal period. The patient has benefited from dietary intervention, but it's not clear if additional treatment would have provided further benefit.	Low
Minet et al. [11]	2020	Case report	France	1	2 mo	Female	Nystagmus, hallucinatory seizures and spasms, truncal hypotonia, decreased visual contact, and pyramidal signs	EEG, MRI, plasma analysis	This study demonstrated neurodevelopmental improvement, significantly fewer seizures, and reduced pipecolic acid as a biomarker of the illness.	Low
Chidambaram et al. [27]	2020	Case report	India	1	9 mo	Female	NR	EEG, MRI, TMS, UGC, genetic analysis	A high level of suspicion for PDE is needed in infants with drug-resistant epilepsy of unknown cause. To identify delayed responders while awaiting further testing, it is recommended to continue pyridoxine therapy for an extended period, even if there is no immediate response.	Low
Wang et al. [28]	2019	Case control	United States	15	4.5 y	NR	NR	Plasma, serum, dried blood spots, urine, and dried urine spots	The concentrations of a-AASA, P6C, AASA-P6C, PA, and a-AAA before and after taking a single oral dose of pyridoxine for the same analyte detected in the same type of sample varied among patients. The mean concentrations increased in almost all the metabolites after taking an oral dose of pyridoxine, with or without statistical significance. However, the metabolites concentrations might increase or decrease among different patients, or in different samples of the same patient, without a regular tendency. There was no statistical correlation between the concentrations before and after taking pyridoxine in the same type of sample for most metabolites.	Medium
Klotz et al. [29]	2017	Case report	Germany	1	1 wk	Female	Episodes of tonic stiffening of the whole body, conspicuous change of breathing rhythm, and mild cyanosis	MRI, genetic test, plasma, urine, and CSF amino acids	VB6 responsiveness is demonstrated by the remarkable seizure response to VB6 therapy and the exacerbation of seizures upon discontinuation of VB6 therapy.	Low
Yuzyuk et al. [13]	2016	Case series	United States	2	NR	1 female, 1 male	NR	MRI, EEG, biochemical, genetic test	The results further support dietary therapies combined with pyridoxine for treating PDE.	Low
Tort et al. [30]	2016	Case report	Spain	1	10 y	Female	Astatic myoclonic epilepsy	Biochemical and genetic test	We emphasize the importance of accurately characterizing biochemical markers to guide the analysis of next-generation sequencing data. High lysine and C10:2-carnitine levels helped identify the second reported case of NADK2 deficiency. Clinical improvement may be attributed to a lysine-restricted diet and PLP administration.	Low
Leganés-Ramos et al. [31]	2016	Case report	Spain	1	16 d	Female	Hyperexcitability with myoclonic seizures	Blood, urine, CSF test	This study demonstrated that preparing a pyridoxine oral solution has been an effective alternative for treating PDE. It has also allowed for dosage adjustment based on the patient's weight in pediatric cases. The patient did not experience any side effects and showed good tolerance to the formula. Currently, she remains asymptomatic.	Low
Mahajnah et al. [14]	2016	Case report	Canada	1	3.5 y	Male	NR	Clinical, biochemical, molecular genetic test	The results showed gross motor delay after 13 mo of age. Tryptophan supplementation was added for mild cerebral serotonin deficiency at the 13th month of therapy, and arginine supplementation was added to further decrease the cerebrospinal fluid (CSF) α-AASA levels at the 26th month of therapy. The CSF α-AASA levels were markedly decreased on this combined therapy.	Low
Tamaura et al. [32]	2015	Case report	Japan	1	23 y	Female	Generalized tonic-clonic seizures	Clinical and genetic tests, EEG	This case highlights the importance of genetic testing for PDE to avoid misdiagnosis and unnecessary withdrawal of pyridoxine.	Low
Coughlin II et al. [33]	2015	Case series	United States	6	3 mo	3 females, 3 males	Hypoglycemia, acidosis, episodes of stiffening with brief jerking, apnea, bilateral temporal lobe hemorrhages, thalamic changes, thrombocytopenic purpura, splenomegaly	EEG, MRI, clinical and genetic test	The study reports the use of triple therapy in a rare condition, showing that early diagnosis and treatment with this new therapy improve cognitive impairment in PDE.	Low
Cirillo et al. [34]	2015	Case series	United States	2	NR	2 female	Hypotonia, excessive jitteriness, myoclonic jerks	EEG, MRI, biochemical markers and gene testing	This study suggests that oral pyridoxine treatment should be continued until biochemical and/or genetic testing confirms the presence or absence of pyridoxine-dependent epilepsy.	Low
Karnebeek et al. [35]	2014	Case series	Canada	6	0–>19	3 male and 3 female	NR	NR	Patients with confirmed ATQ deficiency may be eligible for a lysine-restricted diet as an adjunct treatment unless pyridoxine treatment alone has resolved all symptoms. Lysine restriction should begin early, but the optimal duration is unknown. Monitoring and adherence to recommendations are crucial for ensuring quality care and safety.	Low
Mercimek-Mahmutoglu et al. [36]	2014	Case report	Canada	1	22 mo	Male	NR	Clinical, biochemical, and molecular genetic tests	The levels of CSF α-AASA and CSF pipecolic acid decreased but did not normalize. The patient had a normal neurodevelopmental outcome on a lysine-restricted diet. Despite normal CSF and plasma tryptophan levels and intake, mild CSF serotonin deficiency developed after 1 y of therapy. Stricter lysine restriction may be needed to normalize CSF α-AASA levels, but this could increase the risks associated with the diet.	Low
Mercimek-Mahmutoglu et al. [37]	2014	Case report	Canada	1	12 y	Male	NR	Plasma and CSF sample	L-arginine therapy was well tolerated without any side effects. Cerebrospinal fluid AASA decreased by 57% after 12 mo of therapy. Neuropsychological assessments showed an increase in the general abilities index from 108 to 116, as well as improvements in verbal and motor functioning after 12 mo of therapy.	Low
Oliveira et al. [38]	2013	Case series	Portugal	4	14.75 y	Male	Tonic, myoclonic tonic-clonic, and atonic seizures	MRI, EEG, CTS, biochemical and genetic test	A therapeutic trial with pyridoxine should be conducted in all cases of neonatal, infantile, and childhood refractory epilepsy.	Low
Ware et al. [39]	2013	Case series	Australia	2	21 mo	2 male	Persistent multifocal myoclonic jerks, an eye movement disorder, tongue thrusting	EEG, MRI, clinical, metabolic test	These results indicate that differential responses to pyridoxine and pyridoxal-5′-phosphate treatment cannot be relied upon to diagnose PNPO deficiency.	Low
Karnebeek et al. [40]	2012	Case-series	Switzerland	7	7 wk	6 female, 1 male	Epileptic encephalopathy, hypoglycemia, lactic acidosis	MRI, EEG, biochemical tests, age-appropriate tests and parental observations	The reduction in biomarker levels (measured as the last value before and the first value after the initiation of dietary lysine restriction) ranged from 20% to 67% for plasma pipecolic acid, from 13% to 72% for urinary AASA, 45% for plasma AASA, and 42% for plasma P6C. Improvement in age-appropriate skills was observed in 4 out of 5 patients who showed pre-diet delays, and seizure control was maintained or improved in 6 out of 7 children.	Low
Mishra et al. [41]	2010	Cohort	India	21	5.1 mo	13 males and 8 females	Generalized polyspike, multifocal, generalized spikes, focal	MRI, EEG, serum electrolytes, blood lactate and ammonia, blood gases, and urine-reducing substance	No patient showed normalization of EEG during the “trial.” Two patients (9.5%) responded during the 2 wk of oral treatment, and oral therapy was continued.	Medium
Kuo et al. [42]	2002	Case report	Taiwan	1	9 mo	1 female	Head nodding, myoclonic limb seizures	EEG, MRI, hematologic, and blood biochemistry test	Pyridoxal phosphate should be carefully considered as the drug of choice instead of the more traditional treatment of pyridoxine for patients suspected of pyridoxine-dependent epilepsy. This can help reduce the failure rate and further complications.	Low
Hellström-Westas et al. [43]	2002	Case series	Sweden	2	NR	1 male, 1 female	Tachypnoea, intermittent opisthotonus, downward gaze, multifocal myoclonic seizures	X-ray, CT	One infant with pyridoxine-responsive seizures and another with pyridoxine-dependent seizures had different electroclinical responses on amplitude-integrated EEG monitoring (aEEG) when pyridoxine was administered.	Low
Grillo et al. [44]	2001	Case report	Brazil	1	4 mo	Male	Hypotonia, irregular breathing	EEG, MRI	Using low-dose pyridoxine in multivitamin supplements may conceal pyridoxine dependency, delaying early diagnosis and proper treatment.	Low
Mikati et al. [45]	1990	Case series	United States	6	First week to 14 mo	4 males, 2 females	Partial seizures with or without generalization; myoclonic, atonic seizures	EEG, cranial CT	After stopping vitamin B6, some patients experienced seizures and specific EEG changes, whereas others had persistently abnormal EEG backgrounds and developmental delays.	Low
Baxter et al. [46]	1996	Case series	England	6	7.6	NR	Jitteriness, encephalopathy, hypoxic-ischemic; hepatomegaly, abdominal distension with bilious vomiting	MRI, EEG	The MRI showed structural abnormalities in early-onset cases. The psychometric assessment revealed a specific impairment of expressive verbal ability. An increased dose of pyridoxine was associated with improved IQ in a 1-y prospective study.	Low
Goutières et al. [47]	1985	Case series	France	3	16 mo	1 female, 2 males	Cyanotic, restlessness, loss of appetite, vomiting, irritable behavior	EEG, CTS	The results suggest that all seizure disorders with onset before 18 mo of age should undergo a trial of pyridoxine, regardless of type.	Low
Sokoloff et al. [48]	1959	Case report	United States	1	6 y	1 female	NR	Cerebral blood flow, cerebral metabolic rate, arterial-cerebral venous oxygen difference, cerebral respiratory quotient	This study showed that pyridoxine deficiency could cause seizures due to increased cerebral oxygen consumption from heightened neuronal activity, in addition, a decrease in cerebral metabolic rate would be expected.	Low

Abbreviations: AASA, α-aminoadipic semialdehyde; CSF, cerebral spinal fluid; LRT, lysine reduction therapy; NR, not reported; PDE, pyridoxine-dependent epilepsy; PMH, past medical history; PNPO, pyridoxine phosphate oxidase; TMS, tandem mass spectroscopy; UGC, urine gas chromatography.

### Methodological quality assessment

In this systematic review, we executed a comprehensive quality assessment of various study designs, encompassing cohort studies, case reports, cross-sectional studies, case series, and case-control studies. For case series and case reports, the Newcastle-Ottawa Scale (NOS) was employed [49]. This scale is structured around 3 key domains: comparability, selection, and outcome. Each study was scored based on predefined criteria within these domains, leading to a cumulative score that spans from 0 to 9 points [49]. The scoring criteria included the representativeness of the study population, the appropriateness of the comparison group, the ascertainment of outcome and exposure, and the adjustment for confounding factors. To assess the quality of the chosen case-control and cohort articles, Joanna Briggs Institute's (JBI) tool was utilized [50]. To evaluate and categorize studies utilizing the JBI assessment tools, we analyzed the proportion of affirmative responses for each study in relation to the 8–10 standardized questions pertinent to each study design. A percentage score was derived from the total possible affirmative responses (for example, 8 of 10 affirmative responses equates to an 80% score). A high risk of bias was illustrated by scores between 0% and 33%, whereas scores from 34% to 67% reflected a medium risk. Conversely, scores ranging from 65% to 100% were associated with a low risk of bias [50]. To ensure rigorous application of the NOS and JBI, 2 independent reviewers (SR and HA) evaluated each study. Differences in the evaluations made by the reviewers were addressed through discussions with the corresponding author (SD). The findings from the quality assessment are presented in Table 1. Moreover, Supplemental Tables 1–3 visually represent the quality scores across the various study designs included in our review for both the NOS and JBI tools.

### Data analysis

We organized the study findings based on monotherapy, dual therapy, and triple therapy. In instances where multiple publications utilized the same datasets and we could not pinpoint any overlapping cases, we opted for the publication with the largest sample size. Any inconsistencies among the published versions were noted. We provided a narrative synthesis for each therapy type. When possible, we compared cases of PDE that met the inclusion criteria with control subjects who were epilepsy-free. For visualization of brain regions, we employed the “ggseg” and “ggseg3d” packages in RStudio version 2024.09.0, utilizing R version 4.3.3 [51].

## Result

### Study selection and screening

**FIGURE 2:**
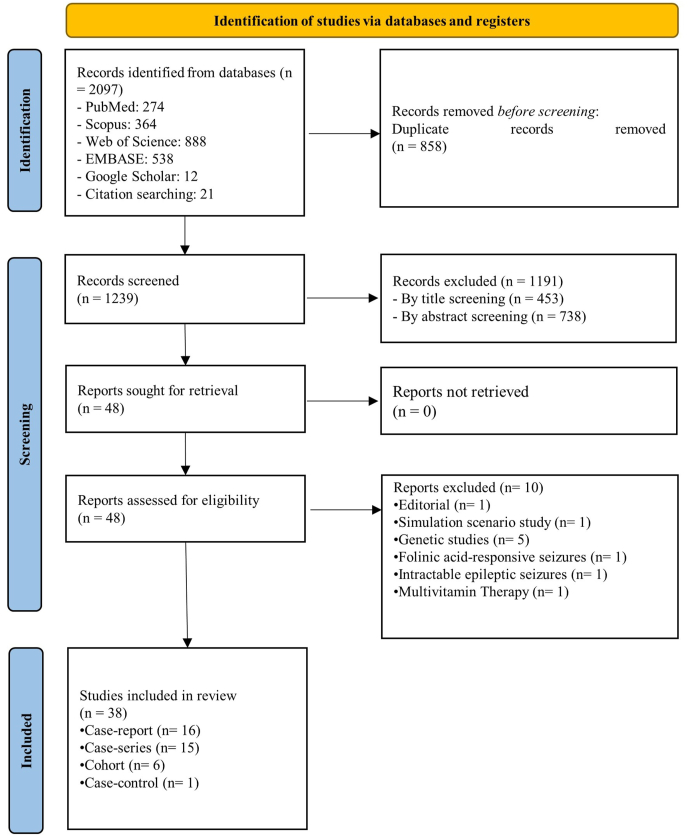
PRISMA flow chart of systematic review.

The process of selecting articles is illustrated in Figure 2. In total, 2097 records were attained from extensive searches across various databases: PubMed (*n* = 274), ISI Web of Science (*n* = 888), Scopus (*n* = 364), Embase (*n* = 538), and Google Scholar (*n* = 12). After the removal of 858 duplicate entries, a total of 1239 studies were chosen for the initial screening of titles and abstracts. Of these, 1191 articles were deemed irrelevant and subsequently excluded from further consideration, resulting in 48 full-text articles designated for further assessment. Ten articles were subsequently excluded based on being Editorials [52], simulation scenario studies [53], genetic studies [[54], [55], [56], [57], [58]], studies related to folinic acid-responsive seizures [59], intractable epileptic seizures [60], and multivitamin therapy [61]. In conclusion, a total of 38 articles were incorporated into the systematic review, comprising 16 case reports [11,12,14,17,24,26,27,[29], [30], [31], [32],^34^,^36^,^37^,^42^,^48^], 15 case series [10,13,34,[18], [19], [20],^25^,^35^,[38], [39], [40],^43^,^45^,^46^,^47^], 6 cohort studies [16,[21], [22], [23],^41^], and 1 case-control study [28].

### Characteristics of the chosen articles

The attributes of the chosen articles are delineated in Table 1. The articles entered were executed between 1959 and 2024 in Italy [16,19], Australia [26,39], Brazil [44], Canada [14,36,36,20,25,35], China [18], England [46], France [11,47], Germany [21,29], India [27,32,41], Japan [32], South Korea [24], Netherlands [22,23], Portugal [38], Spain [30,31], Sweden [43] Switzerland [40], Taiwan [42], and the United States [10,12,13,17,28,34,48,45,62]. The aggregate participant count across the chosen articles totaled 230 individuals, which included 17 from case reports, 59 from case series, 139 from cohort studies, and 15 from a case-control study. The mean age of individuals participating in these selected articles ranged from 1 d to 25 y.

### Methodological quality assessment

The comprehensive quality assessment results, which encompass individual study scores, are presented in Table 1, with detailed scoring information accessed in Supplemental Tables 1–3. Using the NOS tool for case-control and cohort studies, this assessment uncovered 1 study exhibiting a low risk of bias [62], alongside 5 studies categorized with a medium risk of bias [16,21,23,28,41], and 1 study identified as having a high risk of bias [22]. Additionally, employing the JBI tool for case reports and case series revealed that none of the publications were classified as a high or medium risk; instead, all were designated as having a low risk of bias [[10], [11], [12], [13], [14],[17], [18], [19], [20],[24], [25], [26], [27],[29], [30], [31], [32],[34], [35], [36], [37], [38], [39], [40],^42^,[43], [44], [45], [46], [47], [48]].

### Monotherapy

A total of 22 studies have evaluated the efficacy of monotherapy with pyridoxine or its derivatives in the management of PDE [17,27,29,31,32,18,19,20,34,42,48,38,39,[43], [42], [41],^21^,^16^,^28^,^44^]. Most of these studies demonstrated that pyridoxine monotherapy effectively controls seizures in PDE, particularly in cases that are resistant to conventional antiepileptic drugs (AEDs). For instance, Goutières et al. [47] reported immediate seizure cessation after intravenous administration of pyridoxine, which was subsequently maintained through oral supplementation. Similarly, Mikati et al. [45] highlighted significant neurodevelopmental benefits when treatment was initiated promptly after the onset of seizures, thereby emphasizing the significance of early intervention within the first 6 months of life. Grillo et al. [44] noted that even minimal doses of pyridoxine included in multivitamin supplements could control seizures in some instances, although these low doses often resulted in delays in formal diagnosis. Furthermore, Oliveira et al. [38] emphasized the relevance of genetic and biochemical markers, such as elevated urinary AASA, in confirming the diagnosis of PDE and monitoring treatment response. Furthermore, they illustrated that a combination of AEDs and oral pyridoxine ultimately resulted in seizure cessation and normal EEGs, alongside improvements in psychomotor development despite brain imaging revealing atrophy and corpus callosum hypoplasia (Supplemental Figure 1) [38].

However, variability in treatment outcomes was addressed in several studies. Kuo and Wang [42] reported instances where pyridoxine alone was inadequate, with pyridoxal phosphate demonstrating superior efficacy. Hellström-Westas et al. [43] described variations in electroclinical responses during pyridoxine treatment, underscoring the necessity for tailored therapeutic strategies. Another study executed by Mishra et al. [41] identified a subgroup of patients who achieved complete seizure remission through oral pyridoxine monotherapy. These findings underscore the necessity of conducting systematic trials of pyridoxine in cases of early-onset refractory epilepsy. Moreover, Falsaperla et al. [16] provided evidence that delayed initiation of pyridoxine therapy correlates with poorer neurological outcomes. Conversely, early initiation of treatment, within the first 6 months of life, was associated with improved cognitive and developmental trajectories, as observed by Oliveira et al. [38]. Table 2 [17,21,29,32,38,39,43,45,47,48] presents the EEG interpretations from the selected articles, categorized into 3 phases of prepyridoxine administration, postpyridoxine administration, and long-term effects.

**TABLE 2:** EEG interpretation of included studies using pyridoxine as monotherapy.

Study	Prepyridoxine findings	Postpyridoxine findings	Long-term effects
Fortin et al. 2023 [17]	Before pyridoxine administration, the EEG shows a severely abnormal burst-suppression pattern, indicating significant brain dysfunction, often seen in encephalopathy or metabolic issues.	48 h after pyridoxine, the EEG continues to show burst-suppression, though some activity is observed between bursts, suggesting partial improvement but ongoing abnormal brain activity.	By day 5, the EEG shows normal activity with typical neonatal patterns during wakefulness. Mild discontinuity during sleep suggests slight lingering brain dysfunction, though recovery is underway.
Bayat et al. 2022 [21]	Participant 1: moderate background slowing with infrequent trains of delta activity or sharp and slow waves in the prefrontal regions. Participant 5: moderate background slowing with infrequent trains of delta activity or sharp and slow waves in the prefrontal regions. Participant 7: reduced slowing of background activity, with multifocal sharp/spike waves and multifocal bursts of delta activity during relaxed wakefulness.	Participant 1: by day 3, there was a mild progressive reduction in EEG abnormalities. Participant 5: unchanged or accentuation of epileptiform abnormalities (that is, worsening of the original EEG patterns). Participant 7: one hour after intravenous pyridoxine, the EEG showed significant reduction of slowing and disappearance of interictal epileptiform discharges.	Participant 1: 10 wk after pyridoxine, the EEG showed gradual improvement in abnormalities after treatment. Participant 5: 1 mo after pyridoxine, the EEG findings suggest that the abnormalities persisted or worsened after treatment. Participant 7: 10 wk after pyridoxine, the EEG showed a reappearance and worsening of interictal epileptiform discharges.
Klotz et al. 2017 [29]	Discontinuous pattern with multifocal sharp waves, predominantly over the right hemisphere.	The EEG shows a continuous pattern with increased multifocal epileptiform activity during sleep. This indicates that, over time, the epileptiform discharges have become more widespread and persistent.	3 mo after starting pyridoxine, there is a significant improvement in the EEG, showing reduced epileptiform activity and a return to a more regular sleep pattern.
Tamaura et al. 2015 [32]	The EEGs showed a burst-suppression pattern.	On day 24 after birth, an intravenous injection of 70 mg pyridoxine stopped the seizures and normalized the EEGs.	NS
Ware et al. 2013 [39]	The EEG showed intermittent diffuse slowing and irregular generalized epileptiform activity in the encephalopathic state, associated with eye rubbing in the first patient.	In the cerebrospinal fluid chromatograms, the second patient treated with pyridoxal 5′-phosphate had markedly elevated pyridoxamine levels and comparatively low pyridoxine levels.	NS
Oliveira et al. 2013 [38]	At 13 mo, before the pyridoxine trial, the EEG showed bilateral slow-wave activity.	NS	Four months after the second pyridoxine trial, at 19 mo of age, the EEG displayed a well-structured pattern.
Hellström-Westas et al. 2002 [43]	The infant experienced recurrent clinical seizures for the first 3.5 h, accompanied by corresponding electrographic seizure activity, depicted as a "saw-tooth" pattern on the EEG tracing.	Thirteen minutes after the administration of 100 mg pyridoxine through a nasogastric tube, both clinical and electrographic seizures ceased, the EEG background became continuous with a slightly periodic pattern showing low minimum amplitude but normal maximum amplitude, and cyclical changes suggestive of sleep-wake cycling were observed in the EEG background.	NS
Mikati et al. 1990 [45]	The patient, 7 wk old, exhibited bilateral runs of rhythmic 13 Hz sharp and slow-wave complexes during a clinical seizure. The EKG channel displayed rhythmic muscle artifact, indicating ongoing seizure activity.	Three minutes after receiving an intravenous injection of 50 mg pyridoxine, the clinical seizure subsided, the patient was clinically asleep, and the EEG background showed marked suppression on the left side and nearly normal activity on the right side.	NS
Goutières et al.1985 [47]	Patients experienced generalized clonic seizures, characterized by generalized spikes, polyspikes, and high-voltage slow waves.	After the second 220, the seizures start to diminish, and the paroxysmal EEG activity reduces.	After the second 560, the seizures cease completely, and the EEG shows generalized slow waves.
Sokoloff et al. 1959 [48]	NS	After the second 2 and by using 15 mg IV injection of pyridoxine, the EEG activity reverted to the normal pattern.	NS

Abbreviations: EEG, electroencephalogram; IV, intravenous; NS, not specified.

### Dual therapy

The findings from 9 studies investigating the effects of combined therapies utilizing pyridoxine in conjunction with LRD or AS for the management of PDE demonstrate the potential of these dual therapeutic strategies [30,36,37,25,35,40,23,22,62]. These approaches aim to enhance metabolic control and improve neurodevelopmental outcomes by targeting neurotoxic lysine metabolites. Several studies have reported significant biochemical and developmental benefits associated with these combinations. For example, Karnebeek et al. [40] documented that lysine restriction led to a notable decrease in plasma and CSF levels of PA and AASA, with the majority of patients achieving maintained or improved seizure control. Similarly, Mercimek-Mahmutoglu et al. [36] found that L-AS led to decreased AASA levels and enhanced verbal and motor functions, underscoring the potential of metabolic modulation. Coughlin et al. [62] highlighted clinically significant improvements in developmental test scores when LRDs were initiated alongside pyridoxine within the first 6 months of life. Tseng et al. [23] corroborated these findings, reporting superior neurodevelopmental outcomes in siblings who received early combined therapy within the first 6 months of life compared with those who were treated later. The dual therapy was well tolerated across the studies, with no severe adverse events reported. However, Mercimek-Mahmutoglu et al. [37] noted mild serotonin deficiency as a potential side effect of strict lysine restriction, emphasizing the necessity for accurate dietary management and neurotransmitter monitoring. Schmidt et al. [25] explored the antagonistic interaction between arginine and lysine, demonstrating that AS reduces systemic lysine oxidation, thereby mitigating the accumulation of neurotoxic lysine metabolites. In adult patients, Tseng et al. [22] observed variations in neurological and cognitive outcomes, highlighting the need for personalized therapeutic approaches.

### Triple therapy

Seven studies evaluated the efficacy of triple therapy in managing PDE [[10], [11], [12], [13], [14],^24^,^26^]. This therapeutic approach addresses the underlying metabolic derangements associated with PDE while enhancing both clinical and developmental outcomes. The studies collectively demonstrated that triple therapy significantly reduces neurotoxic lysine metabolites, enhances seizure control, and improves neurodevelopmental outcomes. All 7 studies reported good tolerability for triple therapy, with no significant adverse effects noted. For instance, Coughlin et al. reported substantial reductions in CSF and plasma AASA levels. The addition of AS to LRD and pyridoxine resulted in improved motor outcomes in some patients, with early initiation within the first 6 months of life optimizing developmental results [10]. Similarly, Mahajnah et al. [14] highlighted the long-term benefits of triple therapy, including seizure freedom, improved cognitive outcomes, and reduced CSF AASA levels. Although the biomarkers remained elevated in some cases, the therapy was well-tolerated and supported significant developmental progress. Yuzyuk et al. [13] emphasized the clinical and biochemical benefits of triple therapy, demonstrating a strong correlation between reduced plasma lysine levels and enhanced developmental parameters, thereby underscoring the importance of lysine restriction and metabolic monitoring. Kava et al. [26] reinforced these findings by documenting neurological and imaging improvements, such as normalized EEGs and reduced cerebral white matter abnormalities, in a child undergoing triple therapy. Minet et al. [11] demonstrated that combining LRD with pyridoxine and AS led to fewer seizures, enhanced neurodevelopment, and decreased PA levels. Early initiation within the first 6 months of life consistently yielded better outcomes, as supported by Kim et al. [12], who observed significant improvements in the biomarker levels and seizure control when therapy commenced during infancy. Ryu et al. [24] described marked behavioral and cognitive improvements in a young boy with previously intractable seizures, who became seizure-free and capable of following commands. However, residual cognitive challenges persisted, highlighting the need for early intervention to prevent irreversible damage. Mahajnah et al. [14] indicated mild serotonin deficiency associated with lysine restriction, which was effectively managed through tryptophan supplementation. The studies emphasized the importance of nutritional monitoring to prevent potential malnutrition resulting from excessive lysine restriction. Table 3 [[10], [11], [12], [13], [14],^24^,^26^] illustrates the dosages utilized and the ages of individuals receiving triple therapy.

**TABLE 3:** Triple therapy timeline and dosage in accordance with included articles.

Study	Pyridoxine IV (age)	Arginine supplementation (age)	Lysine-restricted diet (age)
Minet et al. 2020 [11]	15 mg/kg/d (3 m–48 m)	150 mg/kg/d (18–48 m)	55-60 mg/kg/d (16–48 m)
Coughlin II et al. 2022 [10]	15–40 mg/kg/d (11 d–3.5 y)	150 – 200 mg/kg/d (99 d–8 y)	NS (28 d–3.5 y)
Mahajnah et al. 2016 [14]	13–44 mg/kg/d (7 m–41 m)	400 mg/kg/d (35–41m)	50 –80 mg/kg/d (10–41 m)
Yuzyuk et al. 2016 [13]	10–20 mg/kg/d (11 d–33 m)	150 mg/kg/d (23–26 m)	NS (20–22 m)
Kava et al. 2020 [26]	100 mg/d (10 d)	NS	45–70 mg/kg/d (14 d–18 m)
Kim et al. 2022 [12]	100 mg/d (12 d)	200 mg/d (12 d)	NS (12 d)
Ryu et al. 2022 [24]	50–300 mg/d (7 y)	120 mg/kg/d	NS

Abbreviations: IV, intravenous; mg, milligram; NS, not specified.

## Discussion

Our systematic review highlights the efficacy of triple therapy—combining pyridoxine, AS, and an LRD—in managing PDE. Findings suggest that although pyridoxine monotherapy effectively addresses seizure activity in most cases, it falls short in mitigating the broader neurodevelopmental challenges associated with the condition. Triple therapy as a promising intervention demonstrated superior outcomes in seizure control, cognitive function, and biomarker regulation. Pyridoxine monotherapy effectively halts seizures by addressing the immediate PLP deficiency but does not mitigate the accumulation of neurotoxic lysine metabolites, which are implicated in long-term cognitive and developmental deficits. Persistent elevations in biomarkers like AASA and P6C have been correlated with suboptimal neurodevelopmental outcomes even in seizure-free individuals. Combining pyridoxine with either LRD or AS offers an enhanced control over lysine metabolism. However, dual therapy does not fully address the multifactorial metabolic derangements in PDE. Evidence suggests that some patients still exhibit elevated biomarker levels and cognitive impairments despite improved seizure control.

To clarify the cognitive outcomes associated with triple therapy (pyridoxine, AS, and LRD) in PDE, we note that treated patients demonstrated improved developmental outcomes compared with the severe intellectual disability often observed in untreated cases [23]. Specifically, Coughlin II et al. [33] reported a 6.9-point increase in developmental testing scores with triple therapy compared with pyridoxine monotherapy, with a significant 21.9-point increase when therapy was initiated within the first 6 mo of life. Similarly, Mercimek-Mahmutoglu et al. [37] observed an increase in the general abilities index from 108 to 116 in a patient on triple therapy, alongside improvements in verbal and motor functioning. These findings indicate that triple therapy mitigates cognitive impairment, with standardized metrics such as developmental testing scores and general abilities indices reflecting clinically meaningful improvements. In contrast, untreated PDE is associated with severe cognitive deficits, with ≤70% of patients exhibiting intellectual disability [22]. These data underscore the importance of early and comprehensive intervention within the first 6 months of life to optimize neurodevelopmental outcomes in PDE.

Triple therapy targets all major facets of PDE pathophysiology, including restoring PLP levels to halt seizures, reducing the lysine-derived production of AASA and P6C, and alleviating systemic and neuronal toxicity by optimizing lysine metabolism. Studies reviewed demonstrated significant reductions in plasma and CSF levels of AASA, P6C, and PA, alongside improvements in motor and cognitive outcomes. Early initiation of triple therapy within the first 6 months consistently yielded the most pronounced benefits, highlighting the importance of addressing PDE's neurotoxic mechanisms promptly.

In congruence with our findings, Coughlin et al. [10] noted optimal results in patients who underwent triple therapy at the early stages of the disease. Any persisting disease symptoms may be linked to early damage indicated by a preliminary MRI before the commencement of treatment, or with severe epilepsy that manifested before the diagnosis was established. Furthermore, the 2021 guidelines recommend starting LRD early in life to achieve the best neurological outcomes [33]. Yet, the authors failed to reach a consensus on the recommended dosage of arginine for pediatric and adolescent populations. Moreover, in a recent follow-up from the global consortium, Dixon et al. [63] recommended to initiate LRD, early in life to achieve the best possible neurological outcomes.

Regarding the duration and potential nutritional consequences of lysine restriction, particularly in infants and adolescents, careful consideration is warranted due to lysine’s role as an essential amino acid critical for growth and development [40]. Studies have reported that strict LRDs can lead to mild reductions in growth parameters, such as height and weight, in some pediatric patients, necessitating close monitoring of anthropometric measures [13]. To mitigate these risks, nutritional management under dietitian supervision is essential to ensure adequate intake of other essential amino acids and micronutrients, with regular assessments of plasma amino acid profiles to prevent deficiencies [36]. Furthermore, short-term LRDs, typically initiated early and adjusted based on biochemical and clinical responses, have been shown to minimize nutritional risks while maintaining therapeutic efficacy in PDE management [33]. Thus, individualized dietary plans, coupled with frequent monitoring of growth, neurodevelopmental outcomes, and biochemical markers, are critical to safely implementing LRDs in infants and adolescents to avoid long-term nutritional compromise.

To address the variability in therapeutic responses, particularly in adolescents (aged 13–18 y) and adults (aged over 18 y), our review indicates that these groups may exhibit less pronounced neurodevelopmental improvements compared with infants and younger children, likely due to prolonged periods of untreated disease [8]. Adolescents often present with residual cognitive deficits and persistent elevations in neurotoxic metabolites, reflecting irreversible neuronal damage from delayed treatment initiation [33]. In adults, the efficacy of triple therapy is further limited by entrenched neurological impairments, although seizure control remains achievable [40]. These findings emphasize the need for age-specific therapeutic strategies, with earlier intervention in infants and children yielding optimal outcomes, whereas adolescents and adults may require tailored approaches to address chronic deficits [63]. Regular monitoring of biochemical markers and neuroimaging is crucial across all age groups to optimize treatment and mitigate long-term complications [10].

### Pathophysiology and the mechanisms addressed by triple therapy

PDE results from biallelic mutations of ALDH7A1 gene, encoding AASAHD, which serves an indispensable function in the catabolic pathway of lysine [64,65]. The absence or dysfunction of AASAHD results in the pathological aggregation of AASA and its cyclic derivative P6C [63,65]. P6C is particularly detrimental as it sequesters PLP, thereby precipitating intractable seizures due to impaired neurotransmitter synthesis, particularly γ-Amino butyric acid (GABA) [[66], [67], [68]].

Pyridoxine supplementation replenishes PLP stores, enabling normal GABA synthesis and restoring inhibitory neurotransmission [67,68]. The rapid cessation of seizures after intravenous pyridoxine highlights its immediate therapeutic impact on the hyperexcitable neuronal state [6,17]. Arginine exerts competitive inhibition on the transport of lysine into the brain and diminishes systemic lysine oxidation [69]. By limiting the availability of lysine, AS indirectly curtails the production of neurotoxic metabolites like AASA and P6C [65]. LRD directly reduces the metabolic flux of lysine catabolism, thereby lowering the systemic burden of neurotoxic metabolites [70]. Together with AS, LRD synergistically diminishes the accumulation of AASA, P6C, and PA, contributing to improved neurodevelopmental outcomes [67,68].

Regarding neurodevelopmental improvements, triple therapy facilitates neurotransmitter biosynthesis like dopamine, GABA, and serotonin by replenishing PLP [65,67,68]. The restoration of balanced excitatory and inhibitory neurotransmission likely underpins the improvements in behavioral and cognitive parameters observed in treated patients [67,70]. The suppression of AASA and P6C prevents further sequestration of PLP and reduces the metabolic stress on neurons [67,68]. The accompanying decline in PA, a neurotoxic marker, further supports the preservation of neuronal integrity [63,65]. Moreover, imaging studies suggest that triple therapy not only stabilizes clinical outcomes but may also contribute to structural normalization, such as improved white matter integrity and reduced cerebral abnormalities [31,63].

### Clinical and practical implications

Early initiation of triple therapy within the first 6 mo may strongly improve the outcomes in patients with PDE. Delayed treatment often results in irreversible cognitive and developmental impairments, emphasizing the need for early diagnostic protocols and genetic testing for suspected PDE cases. Reports indicate minimal adverse effects associated with triple therapy. Mild serotonin deficiency, potentially due to strict lysine restriction, was effectively managed with tryptophan supplementation. These findings underscore the importance of nutritional monitoring to ensure optimal outcomes without compromising patient safety. The variability in responses among older patients or those with prolonged untreated periods underscores the necessity of individualized treatment plans. Incorporating genetic, biochemical, and neuroimaging data could refine therapeutic strategies for PDE.

### Future research directions

Comprehensive longitudinal studies are needed to elucidate the sustained impact of triple therapy on neurodevelopment and quality of life in patients with PDE. Advances in metabolomics may provide novel biomarkers for tracking therapeutic efficacy and tailoring interventions. Lastly, investigating the potential of additional metabolic modulators or neuroprotective agents could further enhance treatment outcomes, particularly in older or treatment-resistant cases.

### Strengths and weaknesses

This systematic review consolidates data from a wide range of study designs, offering robust evidence for the efficacy of triple therapy. Our review provides insights into the biochemical and pathophysiological processes underpinning PDE, enhancing our understanding of therapeutic impacts. The inclusion of practical treatment outcomes, such as seizure control and neurodevelopmental improvements, underscores the real-world applicability of findings. Some limitations must be considered interpreting our results. First, variability in study designs, sample sizes, and outcome measures limits the generalizability of conclusions. Furthermore, few studies explore the prolonged impact of triple therapy, leaving questions about its sustainability and effects into adulthood unanswered. Moreover, case series and case reports dominate the literature, which introduces risk of publication bias and overrepresentation of positive outcomes. Ultimately, the inclusion of predominantly case series and case reports precluded a meta-analysis.

### Conclusion

In conclusion, this review confirms triple therapy as an effective treatment paradigm for managing PDE. By addressing the underlying metabolic disturbances and restoring neurochemical balance, this approach not only controls seizures but also improves neurodevelopmental outcomes. Early diagnosis and intervention within the first 6 months remain pivotal to optimizing patient prognosis. Although triple therapy represents a significant advancement in PDE management, the field stands to benefit from longitudinal studies, the development of novel adjunctive therapies, and the integration of personalized medicine. With continued research and clinical application, the quality of life for individuals with PDE can be markedly improved.

## Author contributions

The authors’ responsibilities were as follows – HA: data extraction, systematic search, preparing the figures, risk of bias assessment, drafting the manuscript, conceptualization, supervision; AJ: systematic search, study selection, drafting the manuscript; MMA: study selection, drafting the manuscript; SR: risk of bias assessment; FB: data extraction; SD: supervision, conceptualization, and critically editing the manuscript; and all authors: read and approved the final manuscript.

## Data availability

This systematic review does not utilize original data. For additional details about the data from the referenced studies, consult the original articles.

## Funding

The authors reported no funding received for this study.

## Conflict of interest

The authors report no conflicts of interest.
