# Supplementary material for: Combination Therapy with Pyridoxine and Arginine Supplementations along with a Lysine-Restricted Diet in Individuals with Pyridoxine-Dependent Epilepsy: A Comprehensive Systematic Review
Source: Curr Dev Nutr. 2025 Jul 8;9(8):107504. doi: 10.1016/j.cdnut.2025.107504 (PMC12341594; doi:10.1016/j.cdnut.2025.107504)
Supplement: multimedia component 1 [file mmc1.docx]

**Supplementary Material**

**Combination Therapy with** **Pyridoxine and Arginine Supplementations Along with a Lysine-restricted Diet in Individuals with Pyridoxine-dependent Epilepsy: A Comprehensive Systematic Review**

Ali Jafari et al

**Supplementary Table 1.** Results of methodological quality assessment of included cohort and case-control studies based on Newcastle-Ottawa Quality Assessment Scale (NOS).

| **Study** | **Selection^1^** | **Comparability^2^** | **Exposure/Outcome^3^** | **Stars Awarded** | **Risk of Bias^4^** |
| --- | --- | --- | --- | --- | --- |
| Falsaperla et al. 2024 | **** | ** | ** | 8 | Medium |
| Tseng et al. 2022 | **** | - | - | 4 | High |
| Tseng et al. 2022 | *** | * | *** | 7 | Medium |
| Coughlin et al. 2022 | **** | ** | *** | 9 | Low |
| Bayat et al. 2022 | *** | * | *** | 7 | Medium |
| Wang et al. 2019 | **** | ** | ** | 8 | Medium |
| Mishra et al. 2010 | *** | * | *** | 7 | Medium |

^1^ Selection: This criterion is rated on a scale from 0 to 4 stars, evaluating the adequacy of the case or cohort definitions, the representativeness of the cases or cohorts, and the methods used for ascertainment and selection of non-exposed or exposed cases.

^2^ Comparability: This criterion is rated on a scale from 0 to 2 stars, assessing the comparability of cases and controls based on the study design or statistical analysis.

^3^ Exposure/Outcome: This criterion is rated on a scale from 0 to 3 stars, focusing on the assessment of the outcome and the ascertainment of exposure.

^4^ A study achieving a maximum rating of 9 stars is classified as having a "Low" risk of bias. Studies receiving 7 or 8 stars are categorized as having a "Medium" risk of bias, while those with 6 stars or fewer are deemed to possess a "High" risk of bias.

**Supplementary Table 2.** Results of methodological quality assessment of included case-reports based on JBI’s Critical Appraisal Tools.

| **Study** | **Q1** | **Q2** | **Q3** | **Q4** | **Q5** | **Q6** | **Q7** | **Q8** | **Score** | **%** |
| --- | --- | --- | --- | --- | --- | --- | --- | --- | --- | --- |
| Fortin et al. 2023 | Yes | Yes | Yes | Yes | Yes | Yes | No | Yes | 7/8 | 87.5 |
| Kim et al. 2022 | Yes | Yes | Yes | Yes | Yes | Yes | No | Yes | 7/8 | 87.5 |
| Ryu et al. 2022 | Yes | Yes | Yes | Yes | Yes | Yes | Yes | Yes | 8/8 | 100 |
| Kava et al. 2020 | Yes | Yes | Yes | Yes | Yes | Yes | No | Yes | 7/8 | 87.5 |
| Minet et al. 2020 | Yes | Yes | Yes | Yes | Yes | Yes | Yes | Yes | 8/8 | 100 |
| Chidambaram et al. 2020 | Yes | Yes | Yes | Yes | Yes | Yes | Yes | Yes | 8/8 | 100 |
| Klotz et al. 2017 | Yes | Yes | Yes | Yes | Yes | Yes | Yes | Yes | 8/8 | 100 |
| Tort et al. 2016 | Yes | Yes | Yes | Yes | Yes | Yes | No | Yes | 7/8 | 87.5 |
| Leganes-Ramos et al. 2016 | Yes | Yes | Yes | Yes | Yes | Yes | No | Yes | 7/8 | 87.5 |
| Mahajnah et al. 2016 | Yes | Yes | Yes | Yes | Yes | Yes | Yes | Yes | 8/8 | 100 |
| Tamaura et al. 2015 | Yes | Yes | Yes | Yes | Yes | Yes | Yes | Yes | 8/8 | 100 |
| Mercimek-Mahmutoglu et al. 2014 | Yes | Yes | Yes | Yes | Yes | Yes | No | Yes | 7/8 | 87.5 |
| Mercimek-Mahmutoglu et al. 2014 | Yes | Yes | Yes | Yes | Yes | Yes | Yes | Yes | 8/8 | 100 |
| Kuo et al. 2002 | Yes | Yes | Yes | Yes | Yes | Yes | No | Yes | 7/8 | 87.5 |
| Grillo et al. 2001 | Yes | Yes | Yes | Yes | Yes | Yes | Yes | Yes | 8/8 | 100 |
| Sokoloff et al. 1959 | Yes | Yes | Yes | Yes | Yes | Yes | No | Yes | 7/8 | 87.5 |

Abbreviations: N/A: not applicable. Q: Question; Q1: Were patient’s demographic characteristics clearly described?; Q2: Was the patient’s history clearly described and presented as a timeline?; Q3: Was the current clinical condition of the patient on presentation clearly described?; Q4: Were diagnostics tests or assessment methods and the results clearly described?; Q5: Was the intervention(s) or treatment procedure(s) clearly described?; Q6: Was the post-intervention clinical condition clearly described?; Q7: Were adverse events (harms) or unanticipated events identified and described?; Q8: Does the case report provide takeaway lessons?

**Supplementary Table 3.** Results of methodological quality assessment of included case-series based on JBI’s Critical Appraisal Tools.

| **Study** | **Q1** | **Q2** | **Q3** | **Q4** | **Q5** | **Q6** | **Q7** | **Q8** | **Q9** | **Q10** | **Score** | **%** |
| --- | --- | --- | --- | --- | --- | --- | --- | --- | --- | --- | --- | --- |
| Chen et al. 2022 | Yes | Yes | Yes | Yes | Yes | Yes | Yes | Yes | Yes | Yes | 10/10 | 100 |
| Amore et al. 2022 | Yes | Yes | Yes | Yes | No | Yes | Yes | Yes | Yes | Yes | 9/10 | 90 |
| Alsubhi et al. 2022 | Yes | Yes | Yes | Yes | No | Yes | Yes | Yes | No | Yes | 8/10 | 80 |
| Schmidt et al. 2020 | Yes | Yes | Yes | No | Yes | Yes | Yes | Yes | Yes | Yes | 9/10 | 90 |
| Yuzyuk et al. 2016 | Yes | Yes | Yes | No | Yes | Yes | Yes | Yes | Yes | Yes | 9/10 | 90 |
| Coughlin II et al. 2015 | Yes | Yes | Yes | Yes | Yes | Yes | Yes | Yes | Yes | Yes | 10/10 | 100 |
| Cirillo et al. 2015 | Yes | Yes | Yes | Yes | Yes | Yes | Yes | Yes | No | Yes | 9/10 | 90 |
| Karnebeek et al. 2014 | Yes | Yes | Yes | Yes | Yes | Unclear | Yes | Yes | No | Yes | 8/9 | 88.8 |
| Oliveira et al. 2013 | Yes | Yes | Yes | Yes | Yes | Yes | Yes | Yes | Yes | Yes | 10/10 | 100 |
| Ware et al. 2013 | Yes | Yes | Yes | Yes | Yes | Yes | Yes | Yes | Yes | Yes | 10/10 | 100 |
| Karnebeek et al. 2012 | Yes | Yes | Yes | Yes | Unclear | Yes | Yes | Yes | Yes | Yes | 9/9 | 100 |
| Hellström‐Westas et al. 2002 | Yes | Yes | Yes | Yes | Yes | Yes | Yes | Yes | Yes | Yes | 10/10 | 100 |
| Mikati et al. 1990 | Yes | Yes | Yes | Yes | No | Unclear | Yes | Yes | Yes | Yes | 8/9 | 88.8 |
| Baxter et al. 1996 | Yes | Yes | Yes | Yes | Yes | Yes | Yes | Yes | Yes | Yes | 10/10 | 100 |
| Goutieres et al. 1985 | Yes | Yes | Yes | Yes | Yes | Unclear | Yes | Yes | No | Yes | 8/9 | 88.8 |

Abbreviations: N/A: not applicable. Q: Question; Q1: Were there clear criteria for inclusion in the case series?; Q2: Was the condition measured in a standard, reliable way for all participants included in the case series?; Q3: Were valid methods used for identification of the condition for all participants included in the case series?; Q4: Did the case series have consecutive inclusion of participants?; Q5: Did the case series have complete inclusion of participants?; Q6: Was there clear reporting of the demographics of the participants in the study?; Q7: Was there clear reporting of clinical information of the participants?; Q8: Were the outcomes or follow up results of cases clearly reported?; Q9: Was there clear reporting of the presenting site(s)/clinic(s) demographic information?; Q10: Was statistical analysis appropriate?


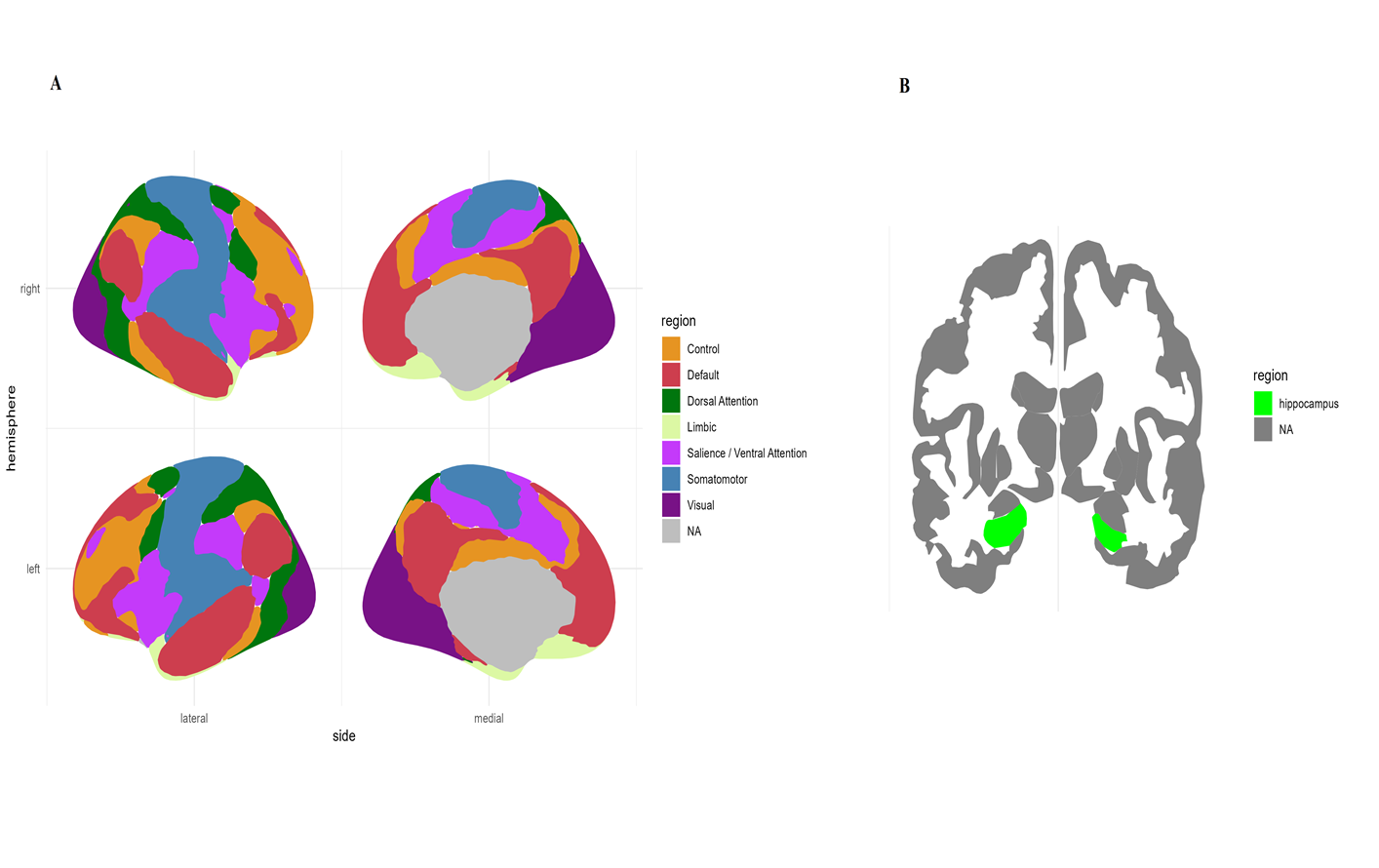
**Supplementary Figure 1.** Visualization of brain regions in patients with PDE. Surface projection model (A) showing lateral and medial views of the left and right hemispheres, with regions color-coded according to the atlas to represent functional networks such as the Default Mode Network (red) and Limbic Network (green). This model highlights findings including global cerebellar atrophy, cortico-subcortical atrophy, bilateral ventriculomegaly, and a thin corpus callosum, as well as white matter abnormalities and gliosis affecting both anterior and posterior periventricular regions. Coronal section model (B) focusing on the bilateral hippocampal regions (green), illustrating mesial temporal sclerosis characterized by atrophy and increased T2 signal intensity in the hippocampi, consistent with structural damage observed in PDE patients.

| **Appendix S1.** Search strategy to find potential eligible studies (November 12, 2024) |
| --- |

**PubMed**

Number of localized studies: 274

|  | **Descriptors** | **Number of studies reached** |
| --- | --- | --- |
| #1 | (((("Lysine"[Mesh]) AND "Diet"[Mesh]) OR "Pyridoxine"[Mesh]) OR "Arginine"[Mesh]) OR (((("Arginine"[Title/Abstract]) OR ("Pyridoxine"[Title/Abstract])) OR ("Vitamin B6"[Title/Abstract])) OR (("Lysine"[Title/Abstract]) AND ("diet"[Title/Abstract]))) | 149814 |
| #2 | ("Epilepsy"[Mesh]) AND (("Pyridoxine"[Title/Abstract]) AND ("Epilepsy"[Title/Abstract])) | 274 |
| #3 | #1 AND #2 | 274 |

**Web of Science**

Number of localized studies: 888

|  | **Descriptors** | **Number of studies reached** |
| --- | --- | --- |
| #1 | TS=("Pyridoxine") OR TS=("Vitamin B6") OR TS=("Arginine") OR TS=("Lysine") AND TS=("Diet") | 277767 |
| #2 | TS=("Pyridoxine") AND TS=("Epilepsy") | 888 |
| #3 | #1 AND #2 | 888 |

**Scopus**

Number of localized studies: 364

|  | **Descriptors** | **Number of studies reached** |
| --- | --- | --- |
| #1 | TITLE-ABS-KEY (Pyridoxine OR “Vitamin B6” OR Arginine OR Lysine AND Diet) | 27,577 |
| #2 | TITLE-ABS-KEY (Pyridoxine AND Epilepsy) | 1,706 |
| #4 | #1 AND #2 | 364 |

**Embase**

Number of localized studies: 538

|  | **Descriptors** | **Number of studies reached** |
| --- | --- | --- |
| #1 | 'Lysine'/exp OR 'Pyridoxine'/exp OR 'Diet'/exp OR 'Arginine'/exp OR 'Pyridoxine':ti,ab OR 'Vitamin B6':ti,ab OR 'Arginine':ti,ab OR 'Lysine':ti,ab OR 'Diet':ti,ab | 1,251,599 |
| #2 | ('Epilepsy'/exp) AND ('Pyridoxine':ti,ab AND 'Epilepsy':ti,ab) | 538 |
| #3 | #1 AND #2 | 538 |
